# Bacteria Associated with Benthic Invertebrates from Extreme Marine Environments: Promising but Underexplored Sources of Biotechnologically Relevant Molecules

**DOI:** 10.3390/md20100617

**Published:** 2022-09-29

**Authors:** Angelina Lo Giudice, Carmen Rizzo

**Affiliations:** 1 Institute of Polar Sciences, National Research Council (CNR.ISP), Spianata S. Raineri 86, 98122 Messina, Italy; 2 Stazione Zoologica Anton Dohrn, National Institute of Biology, Sicily Marine Centre, Department Ecosustainable Marine Biotechnology, Villa Pace, Contrada Porticatello 29, 98167 Messina, Italy

**Keywords:** marine life, extreme habitats, invertebrate–bacteria associations, bioactivity, symbiotic associations

## Abstract

Microbe–invertebrate associations, commonly occurring in nature, play a fundamental role in the life of symbionts, even in hostile habitats, assuming a key importance for both ecological and evolutionary studies and relevance in biotechnology. Extreme environments have emerged as a new frontier in natural product chemistry in the search for novel chemotypes of microbial origin with significant biological activities. However, to date, the main focus has been microbes from sediment and seawater, whereas those associated with biota have received significantly less attention. This review has been therefore conceived to summarize the main information on invertebrate–bacteria associations that are established in extreme marine environments. After a brief overview of currently known extreme marine environments and their main characteristics, a report on the associations between extremophilic microorganisms and macrobenthic organisms in such hostile habitats is provided. The second part of the review deals with biotechnologically relevant bioactive molecules involved in establishing and maintaining symbiotic associations.

## 1. Introduction

The limits of life on Earth are defined by environmental conditions, which include wide ranges of temperature; pH, oxygen, and radiation levels; high pressure and salinity; and limitations in energy, nutrients, and water. However, the definition of “extreme environment” derives from an anthropocentric vision, and extremophilic organisms are metabolically and biochemically active under such extreme conditions, which constitute their normality and to which they are adapted, thus becoming dominant in terms of biomass [1].

Extreme habitats are widely distributed around the globe, and those occurring in the ocean cover more than 50% of Earth’s surface [2], spanning from hydrothermal vents to the Poles and deep-sea. The marine ecosystem includes both stable and unstable extreme environments, possibly inhabited by abundant and endemic organisms. Stable environments, such as polar areas, host organisms that experience the limits of their physiological potential for long periods. Conversely, unstable environments (e.g., hydrothermal vents) undergo stochastic events, leading to the development of peculiar survival strategies by organisms to overcome intermittent and ephemeral conditions [2].

For a long time, environments with parameters that were not on a human-scale were considered a priori as areas impossible to be inhabited. In the 1970s, it became evident that in many extreme and highly harsh environments, life was not precluded in most of its forms (from micro- to macroorganisms). Nowadays, we know that efficient survival strategies are adopted by microbes, as well as invertebrates, that can complete their life cycle under extreme environmental conditions. These include genetic, biochemical, and physiological adaptation processes and are dependent on gene regulation as well as on peculiar structures and functions of cellular components (from proteins to membranes). For this reason, organisms living at the extremes are expected to lead a higher probability of producing structurally unique metabolites, as a reflection of their high genetic diversity and adaptation mechanisms. Extreme environments have emerged as a new frontier in natural product chemistry in the search for novel chemotypes with relevant biological activities [3].

Marine animals live and evolve in a sea of microbes [4]. Symbiotic relationships are widespread in marine ecosystems. Their study is of main concern in aquatic environments for the comprehension of host ecology and evolution. Most benthic invertebrates are associated with epibiotic or endobiotic symbionts, which supply them with critical food resources and may provide the hosts with chemical defense (i.e., production of natural products) against predators (e.g., protozoa, viruses) and the environment (e.g., antibiotics and other chemical toxins, and external pressures). Epibionts can cover almost the entire surface or occur only in a specific part (e.g., appendages, gills) of an animals body, whereas endobionts can be extracellular or intracellular. In the case of sponges, symbiotic microbes can be involved in the sponge’s structural features [5] and in the UV-protection [6,7] of the hosts, as well as in the carbon cycle dynamics by removing important amounts of carbon [8]. Despite this, there is not yet a clear idea of the exact role played by sponge symbionts in the relationship, e.g., [9,10,11]. From a microbial perspective, symbiosis can be considered an ancient survival strategy that furnishes microorganisms with advantages for their proliferation, such as access to a nutrient-rich habitat and a higher environmental stability. In this regard, even if microbial cells typically grow faster in their free-living stage, aggregates confer microbes a higher protection to external stresses (e.g., desiccation, antibiotics, and predation).

Symbiotic partners’ astonishingly close structural, metabolic, and even molecular integrations show the range of the diversity that can result from the fusion of two or more different organisms [12]. By definition, a holobiont is the assemblage of symbionts and their host invertebrates (i.e., a metaorganism). The hologenome hypothesis also contends that holobiont functions as a distinct biological entity and, thus, as a level of selection in evolution because of its hologenome [13]. Major symbionts and the incredibly diverse accompanying microbiota—which have just recently been partially discovered using molecular methods—are both given significant weight in this idea. The holobiont’s distinctive characteristics can be maintained by the intergenerational transmission of both the host DNA and the linked microbiome. This is due to close interactions between microorganisms and their hosts, which affects the fitness of the holobiont (in terms of morphology, development, behavior, physiology, and resistance to disease). For this reason, the theory views the holobiont as a single dynamic entity in which microorganisms contribute genetic information and variety. The microbiome is essential to the adaptability and evolution of the holobiont because it can respond to environmental changes more quickly than the host genome. As a result, mutations in the host genome and/or any associated microbial genomes allow the holobiont to evolve, depending as much on cooperation within the holobiont as on competition with other holobionts. Host and symbionts are often analyzed as a holobiont to reveal adaptive mechanisms of symbiosis [14,15].

If we add to this the fact that microbe–invertebrate associations, commonly present in nature, could play a fundamental role in the life of symbionts, associations in extreme environments assume a key importance for both ecological and evolutionary studies and relevance in biotechnology. In fact, molecules involved in the association in such peculiar ecosystems could possess novel and still undiscovered characteristics; thus, microbe–invertebrate interactions have become potential hot-spots for natural product discovery.

This review has been conceived to summarize the main information on invertebrate–bacteria associations that are established in extreme marine environments. After a brief overview of currently known extreme marine environments and their main characteristics, a report on the associations between extremophilic microorganisms and macrobenthic organisms in such hostile habitats is provided. The second part of the review deals with biotechnologically relevant bioactive molecules involved in establishing and maintaining symbiotic associations.

## 2. Extremophilic Microorganisms

It took time to find out that environments with extreme physico-chemical and climatic conditions were inhabited by phylogenetically different organisms, and just physical or chemical characteristics of a certain environment are used to categorize extremophiles. Therefore, based on temperature values, we can distinguish among *moderate thermophiles* (growing between 50 and 60 °C), *extreme thermophiles* (i.e., microorganisms that can survive and grow at temperatures ranging from 60 to 80 °C), *hyperthermophiles* (i.e., growing above 80 °C), and *psychrophiles* (optimal temperatures below 15 °C). Based on pH values, extremophiles are categorized as *acidophiles* (living at pH < 3) or *alkaliphiles* (living at a pH > 9). The *halophiles* are bacterial species that can survive in environments with high NaCl concentrations (>0.2 M NaCl), whereas *xerophiles* can thrive in environments with low water activity. *Microanaerobes* and *anaerobes*, on the other hand, are bacterial species that can survive in environments with low oxygen tension or in the total absence of oxygen. Piezophiles or hyperpiezophiles are microorganisms that optimally grow at high pressure, i.e., 10–50 MPa and >50 MPa, respectively [16]. Finally, the term *radioresistant* is used to describe bacteria that can survive extreme radiation. The main adaptation mechanisms adopted by extremophiles have been recently reviewed by Somayaji et al. [17].

Several marine ecosystems (e.g., deep-sea hydrothermal vents, cold waters in the Arctic and Antarctica, and deep hypersaline anoxic basins) experience a combination of extreme environmental conditions. Such environments host poly-extremophiles, which are able to simultaneously thrive in different extreme conditions (e.g., thermoacidophilic or haloalkaliphilic bacteria). Some extreme environments are excellent research targets for the study of microbial ecology, evolution, and environmental adaptation due to their reduced biological complexity, general tractability for cultivation-independent molecular analyses, and tight coupling between geochemical and biological processes.

## 3. Extreme Marine Environments and Their Benthic Fauna

**Figure 1:**
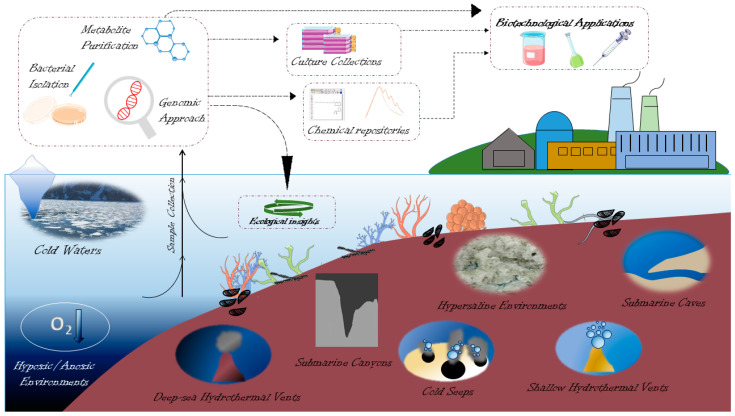
Schematic view of main extreme environments.

A strict categorization of extreme environments is often difficult to achieve. Deep polar regions, for instance, can be included in the deep marine environment, as well as in cold waters. Similarly, submarine canyons can be categorized as cold waters or deep-sea. Overall, extreme marine habitats offer a variety of ecological niches for a large range of organisms, despite their harsh physicochemical constraints. These animals have drawn significant study interest from either a fundamental or practical perspective and have evolved to have distinct coping mechanisms to withstand extreme environmental stresses. This review aims at showcasing the main examples of benthic invertebrates that are adapted to severe environmental conditions and host symbiotic bacteria. Even if extremophilic bacteria are widespread, we have excluded deep hypersaline anoxic basins from the review, due to the fact that such polyextreme ecosystems preclude macrobenthonic life and, therefore, symbiotic association. The main features of macrobenthos-inhabited extreme marine habitats, depicted in Figure 1, are reported in Table 1.

### 3.1. The Deep-Sea

About 95% of the oceans are deeper than 1000 m, but about 50% are deeper than 3000 m (the mean depth of the oceans is 3800 m). Because just 5% of the “deep sea” has been explored thus far, the biotechnological potential of this distinctive ecosystem has not yet been completely exploited [18]. Technically, the deep ocean may be defined as depths beyond the euphotic zone, receiving in the darkness less than 1% of organic matter from photosynthetic primary production [19]. Primary metabolic pathways and, consequently, secondary metabolite production by organisms can be affected by multiple extreme conditions occurring at deep-sea [20]. For instance, pressure increases by 1 atm every 10 m below sea level, light penetration decreases exponentially with depth (with its absence below 250 m), and temperature also decreases with depth (reaching about 2 °C at bathyal depths).

The deep-sea, as the largest biome on Earth, includes a variety of habitats with specific biotic and abiotic features throughout the global ocean, such as deep-sea hydrothermal vents (DSHVs) and cold-seeps (described below), which represent extreme hot-spots of diversity, in evident contrast to the surrounding sea bottom, able to sustain high-pressures, oceanic currents, and variable temperature or pH levels. At DSHVs and cold-seeps, high concentrations of reduced energy sources (e.g., sulfide and methane) are in close proximity to oxidants (e.g., nitrate, sulfate, and oxygen), and food webs are based on chemoautotrophs, deriving energy for their life functions from inorganic chemicals. These habitats act as ‘oases’ in the otherwise nutrient-poor deep sea [21]. In this review, the deep-sea sensu stricto and submarine canyons will be treated among cold water habitats.

#### 3.1.1. Deep-Sea Hydrothermal Vents (DSHVs)

DSHVs were first discovered in 1977 along the mid-ocean ridge of the Galápagos Rift. In volcanic areas, water heated in Earth’s crust by magma may be forced explosively to the surface through rock fissures, thus creating hydrothermal vents. They can be considered as one of the most extreme and dynamic environments on Earth. Temperature is the main factor distinguishing vents in *diffuse flow vents* (from a few degrees above background temperature up to 100  °C), *white smoker vents* (between 100 and 300  °C), and *black smoker vents* (up to 410  °C) [22]. DSHVs are also characterized by extremely steep gradients in physical and chemical gradients between vent fluids and the surrounding seawater [23]. Hydrothermal fluids can be enriched in minerals and transition metals (e.g., aluminum, copper, cobalt, iron, lead, manganese, and zinc), as well as in dissolved gases (including H_2_S, CH_4_, and CO_2_). Under such reducing conditions, Bacteria and Archaea become responsible for a local source of primary production via chemosynthesis in the absence of light, with chemical energy replacing solar energy. The microorganisms therefore sustain an invertebrate biomass higher than that of the surrounding deep waters [24]. Many vent specialist metazoans, such as siboglinid tubeworms, bathymodiolin mussels, and *Kiwa* anomuran crabs, establish nutritionally dependent relationships with chemosynthetic microorganisms, generally Gammaproteobacteria and Campylobacterota members, or also ammonia-oxidizing Thaumarchaeota [25,26,27,28]. The symbiosis of sponges with microorganisms belonging to gammaproteobacterial lineages, such as Methylococcales [29], Marine Methylotrophic Group 2 [30], *Methylohalomonas* [31], and the SUP05 clade [32], is considered a strategy used to survive in the food-limited deep-sea.

According to the report by Chapman et al. [33], megafaunal vent communities throughout the world include 646 taxa from 345 genera, 181 families, and 12 phyla, and they are generally dominated by arthropods, mollusks, and annelid worms, whereas cnidarians and sponges are less represented.

#### 3.1.2. Cold Seeps

Cold seeps (also called cold vents) were discovered in the Gulf of Mexico at 3200 m depth in 1984 [34]. Seep communities with metazoans have been detected from depths of <15 m to >7400 m in the Japan Trench [35]. These areas, found on the oceanic floor worldwide (throughout different depths and latitudes), are fed by subterranean reservoirs of oil and gas from which hydrocarbons are forced through fractured rocks and permeable sediments by pressure gradients into the water column [36]. Cold seeps are generally associated with methane hydrate deposits. Gases rich in sulfide, methane, and bicarbonate have temperatures of around 2 to 4 °C and support the cold seep chemosynthetic communities [37]. Microorganisms use reduced inorganic compounds (e.g., hydrogen sulfide and methane) using oxygen, nitrate, ferric ion, and sulfate as electron acceptors to fix carbon dioxide. The resulting organic carbon is then consumed by higher organisms, leading to the sustenance of an exceptionally high benthic biomass that can weigh up to 500–1000 times more in the seep location than that of the nearby non-seep area [38]. Therefore, cold seeps are one of the most widespread chemosynthetic ecosystems in deep-sea that are densely populated by specialized benthos. Such ecosystems share some common features, for example, the occurrence of bivalves in the families Solemyidae (e.g., genus *Acharax*), Vesicomyidae (e.g., genus *Calyptogena* and *Vesicomya*), and Mytilidae (e.g., genus *Bathymodiolus*), and specialized polychaetes belonging to the family Siboglinidae (e.g., genus *Lamellibrachia, Escarpia*, and *Alaysia*), with Siboglinidae, sponges, and gastropods that are sometimes abundant [35,39,40,41,42,43]. Recently, cold seeps in the Krishna Godavari Basin (East Coast of India) have been demonstrated to also host Gastropoda, Malacostraca, and few species of Echinoidea, Ophiuroidea, and Echiura [44].

### 3.2. Shallow Hydrothermal Vents (SHVs)

Shallow-water hydrothermal vents (<200 m in depth; SHVs) are widespread and generally associated with active submarine volcanism. They can be considered as intermediate environments between deep-sea and terrestrial hydrothermal systems. Fluid temperature ranges between 10 and 119 °C [45]. High concentrations of compounds and metal elements involved in (bio)geochemical cycles (e.g., carbon dioxide, sulfur dioxide, hydrogen sulfide, methane, hydrogen, and iron) are common to several SHVs [46]. pH is seldom acidic at SHVs. Differently from DSHVs, thanks to light availability, primary production is also based on photosynthesis (due to the occurrence of photoautotrophs, e.g., diatoms and algal mats that are absent in DSHV communities).

As for DSHVs, the occurrence of benthic organisms in SHVs is strongly related to the chemistry of volcanic fluids that outflow from the bottom. In SHVs, the acid–base balance is the factor that mainly dominates biodiversity rate, determined by a sort of selection of organisms that live in the surrounding non-vent areas, and have characteristics of adaptability to the vent site. In these areas, calcifying species are less present, favoring the occurrence of encrusting species and the establishment of very simplified benthic communities [47]. The establishment of opportunistic species, including filter feeders, is due to their capacity to take advantage of the increased primary production levels with consequent higher suspended organic matter concentration.

The main taxa structuring the benthic community are generally Mollusca, Sipuncula, Polychaeta, and Crustacea. Some species highly tolerant to extreme conditions of temperature and sulfide concentration, such as the nassariid gastropod *Tritia cuvierii*, sediment-dwelling polychaete *Capitella capitata*, and nassariid *Tritia neritea* and *Nassarius mutabili,* which have been observed in several hydrothermal systems of the Mediterranean area and are considered dominant in macrofaunal communities [48,49]. Species such as the polychaete *Platynereis massiliensis* [50,51] and amphipod *Microdeutopus sporadhi* [52] were recorded in the hydrothermal vents of Ischia Island, while the tube-dweller amphipod *Ampelisca ledoyeri* was near Panarea Island [53].

Chemosynthetic symbiosis at shallow vents has been generally reported with bivalve and polychaete hosts [54]. Very interesting are the peripheral areas of hydrothermal vents, where many individuals of filter-feeding taxa such as serpulid worms and sponges benefit from the availability of hard substrata and the high amount of suspended organic matter. Sponges retrieved near hydrothermal vent systems host communities of sulfide and methane-oxidizing bacteria and relies on such chemosynthetic symbionts [31,32,55].

### 3.3. Cold Water Habitats

Throughout the entire year, almost 85% of the biosphere is exposed to temperatures below 5 °C. Cold ecosystems (both marine and terrestrial) are highly diverse and include bathy- and abysso-pelagic zones, permafrost, sea-ice, glaciers, and to a lesser extent cold soils, groundwater, deserts, lakes, and shallow underground regions. Desiccation, osmotic stress, ice cover, high salinity, low biochemical activity (caused by low temperatures and the Q10 effect, which measures how quickly a biological system changes when the temperature rises or falls by 10 °C), limited nutrient availability, harmful solar radiation, and a highly variable photoperiod (from no light at all to continuous light during a 24-h period) are some of the main characteristics, alone or in combination, that can be found in cold habitats. Therefore, it is not surprising that macroorganisms and microbes living in these severe settings have developed a variety of adaptive strategies to help them survive and thrive.

In the marine realm, other than the polar deep-sea, cold-water habitats inhabited by benthic invertebrates include the temperate and tropical deep-sea, which, below the thermocline, is a nearly constant 4 °C [56].

#### 3.3.1. Polar Marine Environments

In shallow water (less than 1000 m), organisms experience ice at some point in their annual cycle, e.g., in the Southern Hemisphere. Due to the unpredictability in the extent of winter sea ice, it is more difficult to identify Northern shallow-water habitats that encounter seasonal ice [57]. Temperatures below 0 °C are primarily what define polar marine ecosystems. Arctic surface seawater has a temperature range of 0 and +10 °C, while Antarctic surface seawater has an annual range of −1.86 °C to +0.3 °C. Porifera, Cnidaria (including orders Actiniaria, Gorgonaria, Alcyonacea, Pennatulacea, and class Hydrozoa), Bryozoa, Brachiopoda, and Anellida (class Polychaeta) are common components of Antarctic benthic communities, with some taxa that frequently dominate at local scale [58,59,60]. Oleszczuk et al. [61] reported on a total of 271 macrofauna taxa in the Arctic Ocean, including Svalbard fjords, the Barents Sea, and the Eurasian marginal ice zone. Overall, Annelida and Mollusca (55 and 33%, respectively) were the most abundant taxa, followed at a lesser extent by Arthropoda, Sipuncula, and Echinodermata (2–7%). Nemertea, Tunicata, Porifera, Cephalorhyncha, Cnidaria, Entoprocta, Brachiopoda, and Phoronida contributed less than 1% to the total macrofauna community.

#### 3.3.2. Deep-Sea and Submarine Canyons

Deep-sea benthic mega- and macrofauna possess an extraordinarily high level of diversity, although generally not abundant. We could distinguish between soft-bottom (mainly consisting of echinoderms, anemones, polychaetes, bivalve molluscs, diverse crustacea) and hard-bottom deep-sea fauna (dominated by sponges and cnidarians). Most importantly, deep-sea fauna comprise a high number of rare and novel species, often endemic to the deep-sea. The anthozoan orders Scleractinia (stony corals), Octocorallia (soft corals), Anthipatharia (black corals), and the hydrozoan family Stylasteridae (hydrocorals) all include cold-water corals, typically azooxanthellate filter-feeders, meaning they lack symbiotic algae [62]. Both colonial and solitary species are found in water depths down to 5000 m [63]. The most common scleractinian coral, *Lophelia pertusa*, occurs in waters between 4 and 12 °C.

Major geomorphic features of continental margins all over the world include submarine canyons, with more than 9000 large canyons covering 11.2% of continental slopes globally [64]. Canyons have a steep, complex topography that influences current patterns and supports a variety of ecosystems, including rocky walls and outcrops to soft sediment [65]. From the productive coastal zone, these geomorphologic features serve as preferential particle-transport channels down continental slopes to the more stable deep seafloor [66]. In fact, canyons can act as sediment and organic matter traps. Typical canyon-related processes, such as locally enhanced internal tides and focused downslope organic carbon transport, provide favorable environmental conditions (current regime, food input) to sustain the resident communities. In particular, canyon habitats provide refuge sites for marine life, including suspension and filter feeders such as cold-water corals and sponge fields [67,68]. Canyons can be a significant source of genetic resources and chemical compounds due to their biodiversity richness [69]. Substrate heterogeneity is a major contributor to the extraordinarily diversified faunal assemblages seen in submarine canyons, along with currents and topography [65,70], with species that are restricted to either hard or soft substrata. Huvenne et al. [67] observed the occurrence of cold-water corals in water depths ranging from 880 to 3300 m in a submarine canyon. The soft coral *Anthomastus* sp., scleractinian coral *L. pertusa,* and octocorals *Primnoa* sp., *Acanthogorgia* sp., and *Acanella* sp. dominated the community. Authors have demonstrated that deep-sea canyons have the potential to serve as natural havens for faunal communities that are vulnerable to human disturbance and to play a critical role as larvae supplies for the recolonization of damaged areas elsewhere on the margin. High densities of gorgonians, pennatulids, and mussels have been also observed. A total 810 macrofaunal taxa belonging to 11 phyla were identified in the Avilés Canyon, southern Bay of Biscay, NE Atlantic, Spain [71]. The macrobenthos mainly included polychaetes (with the only class of Annelida) followed by molluscs, cnidarians, arthropods, and echinoderms. Assemblages between 378 and 1100 m depth are of special interest due to the occurrence of reef-forming corals *L. pertusa* and *Madrepora oculata*, structuring the entire macrobenthic community.

### 3.4. Other Extreme Environments

Our planet hosts a far greater number of different extreme ecosystems than one might imagine, all with very different features. Some of them still remain largely underexplored or completely unexplored, most of the time due to the difficulties in reaching the sites. Despite being highly interesting, few studies are available on these extreme ecosystems, and to the best of our knowledge, symbiotic relationships have been not yet reported. For this reason, in this section, all the environments considered for all intents and purposes as “extreme”, but currently still representing a dark matter in terms of microbe–invertebrate associations, are described.

#### 3.4.1. Hypersaline Habitats

Hypersaline environments are present in all continents, including coastal lagoons, salt and soda lakes, briny pools, and brine channels in sea-ice. To be classified as hypersaline, an environment should have a high salinity level (superior to that of seawater, 34–130 PSU) [72] and could be also saturated. Hugoni et al. [73] distinguished thalassohaline systems, namely, hypersaline environments naturally occurring and deriving from seawater rich in sodium chloride, and athalassohaline systems, which do not have marine origin and therefore contain different ions, such as potassium, magnesium, sodium, and carbonate (i.e., soda lakes). Finally, saltern crystallizer ponds are artificial basins aimed at producing salt [74]. Some examples of marine hypersaline systems are found in the Mediterranean Sea, Dead Sea, and Red Sea [75], but several non-marine systems have also been described, i.e., alkaline soda lakes of Egypt (e.g., Wadi Natrun), soda lakes of Antarctica, and Big Soda Lake and Mono Lake in California. One of the saltiest aquatic environments on Earth is represented by the system of hypersaline lakes of McMurdo Dry Valleys in Antarctica. They are considered extreme due to the stress imposed to cells through the strong salinity gradient between intra- and extracellular environment. Organisms inhabiting hypersaline environments have to cope not only with high salinity levels, but also with factors such as high UV irradiance, low nutrient and oxygen availability, alkalinity and possible occurrence of toxic compounds.

Most of the studies concerning benthic organisms adapted to live in hypersaline environments are focused on lagoon or estuarine environments, close to the sea, and on the benthic meiofauna. Among benthic invertebrates, many nematodes are adapted to hypersaline environments [76], and assemblages of foraminiferans and ostracods have been observed in saltpans, hypersaline lagoons, and salt lakes [77], also in the areas of the Red Sea [78].

#### 3.4.2. Marine Caves

Underwater caves are peculiar marine environments commonly distributed among shallow water systems but also considered as extreme areas [79]. The strong oligotrophy (i.e., low concentrations or absence of nutrients), in addition to the limited photosynthetic processes, low water circulation, and salinity gradient in anchialine caves [80] are among the factors that allow them to be counted among habitats with harsh environmental conditions. In some cases, living conditions are made more drastic by high sulfide concentrations and deoxygenated conditions [81]. Underwater caves are very heterogeneous environments in terms of geological conformation and origin and can host highly diversified habitats. In caves of volcanic origin, hydrogen sulfide occurs at concentrations of >300 μM [68]. Both darkness and low hydrodynamic regime establish conditions similar to those occurring at deep-sea. Moreover, some caves are also characterized by a cold thermal regime [82].

The information on living organisms in submarine caves is really fragmentary and incomplete. It has been reported a conspicuous meiofauna benthic component [2,83], and some studies are also available on the predominant macrofauna members, i.e., sponges or bryozoans, e.g., [84,85].

#### 3.4.3. Hypoxic and Anoxic Environments

Hypoxic environments are typically characterized by very low oxygen concentration and can be found in both shallow and deep marine waters. In shallow areas, they often occur due to low water circulation or to human activities, which, by increasing the input of nutrients, stimulates biomass production and consequent oxygen consumption by microbial activities, resulting in eutrophication conditions [86]. Differently, in deep water areas, hypoxic or anoxic conditions could occur naturally in the oxygen minimum zones (OMZs) [87] or could become oxygen-deprived as a result of redox imbalances in bottom water and sediment pore water [88].

When anoxia (i.e., absence of oxygen) is not a temporal condition but a long-term event, or when it is a persistent condition, as in the Black Sea [89], only some invertebrates are able to cope with the external conditions, and they are mainly represented by really small animals, i.e., nematodes, gastrotrichs, and gnathostomulids, that are able to live in the absence of oxygen and presence of sulfidic elements. For this reason, no cases of invertebrate–microbe symbiosis have been described until now. However, it has been reported that some class of benthic animals, namely, hydroid cnidarians, anemones, and gastropods, are tolerant to hypoxic conditions [90,91]. This suggests that possible associations with microorganisms are not to be excluded in such environments.

## 4. Invertebrates from Extreme Benthic Habitats as Hosts of Microbial Communities

As complex and mutually beneficial interactions, symbiotic associations are also common in extreme and inhospitable marine habitats, where they assume a crucial importance due to highly dynamic and sometimes challenging environments. The following paragraphs are devoted to showcase some examples of association (seldom obligate) between microorganisms and benthic invertebrates in marine extreme environments. Despite the great diversity of macrobenthos in extreme marine environments, to date, the associated bacterial communities, along with the biotechnological potential of symbiosis-involved biomolecules they produce, have been mainly analyzed for few taxa (e.g., Porifera, Cnidaria, and Annelida), as described in the following paragraphs.

### 4.1. Deep-Sea Hot-Spots of Biodiversity

#### 4.1.1. Deep-Sea Hydrothermal Vents

At DSHVs life is sustained by chemosynthetic bacteria, often living in an intimate symbiosis with animals [92]; reviewed in ref. [54]. In fact, the association between invertebrates (e.g., vestimentiferan tubeworms, bivalve mollusks, provannid gastropods, alvinellid polychaete) and chemolithoautotrophic symbionts has been reported in various hydrothermal niches.

Association with chemolithotrophic bacteria was proven for different categories of crustacean, i.e., the shrimp *Rimicaris exoculata* [93,94,95] and the crabs *Kiwa* spp. and *Shinkaia crosnieri* [96]. In these two latter cases, a site specificity within the host’s organs, along with a pivotal role in nutrient supply [97] and detoxification [91], was observed.

It has been demonstrated that nearly all invertebrates assume a large amount of fixed carbon and nitrogen from microbial symbionts, generally dominated by sulfide-oxidizing bacteria. Among them, Campylobacterota have been demonstrated to dominate in the symbiotic association with metazoans at DSHVs. Campylobacterota are versatile chemoautotrophs capable of the oxidation of H_2_ and sulfur compounds coupled with the reduction of oxygen and nitrate.

The polychaete annelid *Alvinella pompejana*, also known as the Pompeii worm or volcano worm, has been well studied in this regard [98,99,100,101]. It does not harbor endosymbionts, but microbial communities colonize the inner part of its tube and are attached to appendages at the back of the animal [102]. The associated microflora includes a multispecies complex of 12 to 15 phylotypes, with Campylobacterota as the dominant group (>98%) [103]. Symbionts are involved not only in the nutrition of the host but also in the detoxification of sulfide and heavy metals [104]. Tasiemski et al. [105] reported on the discovery of the first antimicrobial peptide, called alvinellacin, produced by a symbiotic animal, namely *A. pompejana*, to shape and control the worm’s epibiotic microflora. Vestimentiferan tubeworms, such as the giant gut-less *Riftia pachyptila*, derive their nutrition from the cultivation of sulfur-oxidizing bacteria within the trophosome, a specialized organ [106]. A horizontal transfer of symbionts, through uptake from the environment, occurs upon settlement of the larvae [107]. As for *R. pachyptila*, the giant clam *Calyptogena magnifica* is extremely nutritionally dependent on its chemoautotrophic endosymbionts, which are transmitted vertically [108].

Among vent fauna, mussels and gastropods also host a wide range of endosymbionts. For example, the bacterial community associated with *Helicoradomenia* sp. (Mollusca, Aplacophora), collected at 2500 m depth, included Alpha- and Gammaproteobacteria [109]. Gammaproteobacteria (a sulfur oxidizer and a methane oxidizer) live in symbiosis with the hydrothermal vent mussels *Bathymodiolus azoricus* and *B. puteoserpentis*. Symbionts are housed in the so-called bacteriocytes, i.e., vacuoles within specialized gill epithelial cells [110]. Thioautotrophic symbionts of the deep-sea sponge *Characella* sp. were strongly related to those of *Bathymodiolus* spp. [111]. Similarly, a dual symbiosis occurs in the vestimentiferan tubeworm *Lamellibrachia anaximandri* from Marsili Seamount, hosting two gammaproteobacterial symbiont phylotypes (autotrophic sulfur oxidizers) [112]. Such types of multiple interactions are generally less stable due to the competition among symbionts but could become stable and advantageous in case of resource partitioning between the symbionts, or if redundant metabolic capabilities can be shaped to cope different or fluctuating environmental conditions, typical of marine vent systems. Hydrogen oxidation in addition to sulfide oxidation was observed as additional metabolic capacity in symbionts of *L. anaximandri* from the Palinuro vent. The possibility for one of the symbionts to use another carbon source could reduce competition by stabilizing multiple symbioses.

Other invertebrates host multiple symbionts, gaining the possibility to shift among available geofuels, highlighting niche adaptation in geologically complex sites. For example, the gastropod *Alviniconcha* sp. hosts three symbionts (i.e., two Gammaproteobacteria and one Campylobacterota) in its gills [113]. The Campylobacterota symbiont dominated where the higher H_2_ and H_2_S concentrations occurred in the vent, while the Gammaproteobacteria dominated at decreasing concentrations of both electron donors in the fluids. A dominance of bacteria involved in sulfur cycling was also observed in association with the esophageal gland of the scaly-foot snail *Chrysomallon squamiferum* [113].

#### 4.1.2. Cold Seeps

Animal-microbe interactions at cold seeps are complex and include symbiosis, heterotrophic nutrition, geochemical feedbacks and habitat structure [35]. Certain invertebrates perform symbiosis with chemosynthetic bacteria, whereas some gastropods feed on bacteria and other animals. Tube worms, mussels, and clams can reach very large sizes at cold seeps (up to 2 m, 36 cm, and 18.6 cm, respectively), as a result of symbiont-supported chemoautotrophic nutrition [35]. Such species host either sulfide-oxidizing or methanotrophic symbionts or both. Methanotrophic bacteria have been also reported in symbiosis with the carnivorous, non-filter-feeding sponge *Cladorhiza methanophila* [114,115]. Medina-Silva et al. [116] first reported on epibiont microbial communities found in close association with the chitin tubes of the cold seep vestimentiferan *Escarpia* sp. Pirellulaceae (phylum Planctomycetes), and Methylococcales (phylum Proteobacteria) dominated the worm-associated microbial community. Members of the family Pirellulaceae (phylum Planctomycetes) have previously been reported from marine sponges [117] and are implicated in aerobic ammonia-oxidation. The external surface of the tube worm constitutes an important interface with water and sediment and promotes the establishment of specific microbial communities. This was also observed for *Riftia pachyptila* [118] and *Lamellibrachia* spp. [119]. The tube worm *Paraescarpia echinospica*, collected from a cold-seep area situated on the northern continental slope of the South China Sea (water depth of 1147 m), presumably harbored a single genotype of bacterial endosymbiont. By employing different virulence factors as digestive enzymes, the bacterial symbionts may be able to break down host proteins and exploit host cells as a source of nutrients [120].

The microbial communities hosted by the sponges *Hymedesmia (Stylopus) methanophila* and *Iophon methanophila* from asphalt seeps at Campeche Knolls at 2900–3100 m depth in the southern Gulf of Mexico were characterized [30]. These sites show asphalt flows, oil seepage, gas hydrates, and gas venting [121]. The authors observed the dominance of methane-oxidizing bacteria. Each sponge species hosted specific methane-oxidizing bacteria genotypes, suggesting that the recognition and selection mechanisms, underlying the potential specificity of this symbiosis, exist. Proteobacteria (representing 94.8–99.9% the microbial community) included chemoautotrophic sulfur-oxidizing bacteria and hydrocarbon-degrading *Cycloclasticus* symbionts, in line with the hydrocarbon-rich site.

### 4.2. Shallow Hydrothermal Vents

**Figure 2:**
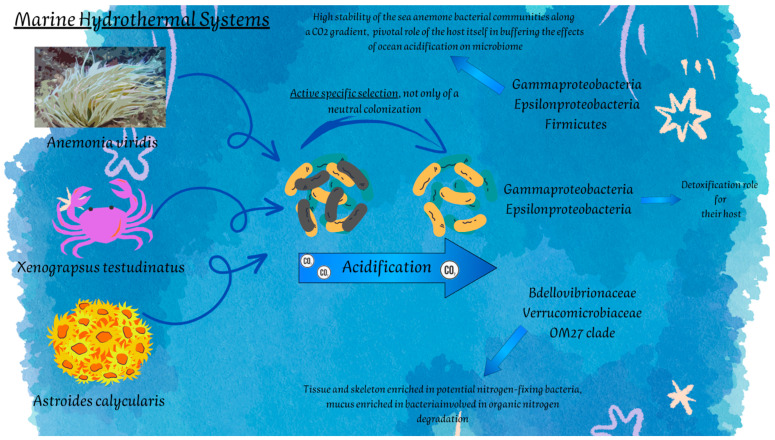
Representative symbiotic relationships at hydrothermal vent system.

Shallow hydrothermal vent benthic inhabitants are based on the populations of chemosynthetic symbiotic bacteria which provide energy and organic carbon [25]. These animals have to combine the uptake of reducing chemicals (i.e., sulfide, hydrogen gas, and methane) from hydrothermal fluids for their chemosynthetic symbionts with their survival strategies under harsh environmental conditions (i.e., thermal stress, low oxygenation rate, high levels of toxic sulfide and heavy metals). The main benthic invertebrates living shallow systems are represented by meiofauna organisms, i.e., copepods and nematodes, especially the family Onchalaimidae [122,123,124], and microbial associations with them have also been described [125], mainly composed of sulfur-oxidizing bacteria affiliated to Campylobacterota and Gammaproteobacteria [125]. However, relationships with macrofaunal benthos are also documented (Figure 2).

The ecological dynamics are based on different mechanisms in DSHS and SHVs, so that different food-webs occur. The shallow systems are not characterized by a chemolithoautotrophic food-web and are moreover less rich in biodiversity than deep systems, thus favoring organisms capable of implementing opportunistic lifestyles. This means that the nature of interspecific interactions is also different. Yang and colleagues [126] investigated the bacterial communities associated with the crab *Xenograpsus testudinatus*, a vent-endemic dominant crustacean living near sulfur-rich hydrothermal vents in Taiwan. Interestingly, the study put efforts in characterizing the bacterial symbionts in different tissues and organs of the individual, by demonstrating the dominance of Gammaproteobacteria and Campylobacterota and strong host- and potential organ-specificities, in support of a trophic symbiotic relationship. In addition to this, and similarly to what was found in deep hydrothermal systems, Gammaproteobacteria and Campylobacterota are probably also involved in a detoxification role for their host, as water around vents in Kueishan Island are proven to be toxic, as they are rich in carbon dioxide, nitrogen, oxygen, sulfur dioxide, hydrogen sulfide [127], and metals, including Mg^2+^, Ca^2+^, Fe^2+^, Cu^2+^, Al^3+^, and Mn^3+^ [128,129].

The microbiome of one sponge, provisionally identified as *Hymeniacidon* novo spec., sampled in Taiwanese hydrothermal vents, resulted in being mainly dominated by Proteobacteria members, specifically Alpha- and Gammaproteobacteria. The predictive analysis also evidenced a high representation of some metabolic pathways, i.e., DNA repair and recombination proteins, chromosomes, DNA replication proteins, homologous recombination, mismatch repair, and DNA replication, in addition to those related to the amino acid metabolism [130]. Recently, a culture-dependent approach was employed to isolate bacteria from different macrobenthic individuals of the shallow hydrothermal vent site in Eyjafjörður, Iceland, including sea anemones, ascidians, macroalgae, and one nudibranch [131]. Actinobacteria (mainly *Streptomyces* spp.) and bacilli, together with Alpha- and Gammaproteobacteria, were identified as the dominant phylotypes and were investigated for their bioactivities (see Section 5.1.1).

Microbial communities associated with sea anemones of different shallow hydrothermal systems have also been studied and related to the acidification processes and consequent pH gradient, by providing interesting ecological insights. Muller et al. [132] characterized the microbial communities of the two anemones, *Anemonia viridis* and *Actinia equina*, living in the volcanic vent system at Levante Bay, Vulcano Island, Italy. As a first observation, the authors reported differences between the two species in the Gammaproteobacteria and Campylobacterota abundances, with Campylobacterota that were more abundant in all *A. equina* samples and mainly represented by sulfide oxidizing bacteria (i.e., *Sulfurimonas*, *Helicobacter*, *Sulfurovum*, and *Sulfospirillum*). Differently, Meron et al. [133] found a predominance of Gammaproteobacteria and Firmicutes in *A. viridis* of Ischia Island, Italy, and a lower Alphaproteobacteria composition than those retrieved in Vulcano sea anemones. However, the highly interesting aspect pointed out by Muller et al. [132] was the high stability of the sea anemone bacterial communities along a CO_2_ gradient, suggesting a pivotal role of the host itself in buffering the effects of ocean acidification on microbiome and in shaping the structure of associated bacterial populations, as proven by taxonomic differences between sea anemone species.

By a similar approach, Biagi et al. [134] investigated the bacterial communities associated with different anatomic compartments of the coral *Astroides calycularis* in individuals inhabiting a volcanic CO_2_ vent in Ischia Island (Naples, Italy) and in non-acidified sites at the same island. Differences in the taxonomic composition of microbial symbionts in acidified and non-acidified habitats were detected mainly in relation to bacterial groups involved in nitrogen cycle in benthic environments. While tissues and the skeleton were rich in bacterial groups involved in nitrogen-fixation processes, the mucus was mainly colonized by bacteria able to degrade organic nitrogen (i.e., Bdellovibrionaceae, Verrucomicrobiaceae). These findings further confirm that the establishment of microbiomes in the host is often the result of an active specific selection, not only of a neutral colonization.

### 4.3. Cold Marine Habitats

#### 4.3.1. Antarctic Marine Environments

In the last two decades, marine Porifera from Antarctica have been intensely investigated for the associated prokaryotic communities.

Culture-independent methods have been applied to different sponge species to investigate the whole community, collected all around Antarctica, at sites in McMurdo Sound and Terra Nova Bay (Ross Sea, Antarctica), Antarctic Peninsula, South Shetland Islands, and Palmer Archipelago (e.g., [135,136,137,138,139,140,141,142,143,144,145,146]). Webster et al. [135] first studied Antarctic-sponge-associated prokaryotic communities. Their results highlighted that these sponges hosted both species-specific and not specialized associated bacteria (i.e., sequences that were closely related to phylotypes previously detected in Antarctic seawater and sea-ice). The first observations by Webster and coworkers [135] were then confirmed by several investigations applying high-through-put sequencing technologies of ribosomal genes, even if the studies were carried out in diverse Antarctic sites by collecting different sponge species. Rodríguez-Marconi et al. [136] highlighted that Antarctic-sponge-associated microbial communities differed at the phylum level from those known for temperate and tropical ecosystems. The assessment of host-specificity was the main aim of recent works by Cárdenas et al. [138], Sacristán-Soriano [142], and Happel et al. [143], who found a high similarity among the microbiota of cold-water sponge species collected from different Antarctic sites. Cristi et al. [145] reported a high degree of specificity of bacterial symbiotic community in *Hymeniacidon torquata*, suggesting reduced presence of transient bacteria. Overall, Proteobacteria (Alpha- and Gammaproteobacteria) generally predominate in Antarctic-sponge-associated bacterial communities, followed by Bacteroidetes. Some sponge species also host Actinobacteria and Firmicutes (e.g., [137,140,141,146]).

Moreno-Pino et al. [140] and Papale et al. [141] combined a microbial community composition analysis with the metabolic potential of microbiomes to individuate those functions possibly involved in the establishment of symbiosis, nutrient exchange, and sponge holobiont survival. Functional analyses revealed that sponge-associated microbial communities were enriched in functions related to the symbiotic lifestyle (e.g., CRISPR system, Eukaryotic-like proteins, and transposases), along with functions involved in nutrient cycling (especially nitrogen cycling and carbon fixation) [140]. The metabolic profiles of sponge-associated microbial communities highlighted pathways related to the biosynthesis of antibiotics, quorum sensing, and degradation or toxic compounds [141]. Taken together, these results highlighted the metabolic potential of Antarctic sponge microbiomes in being involved in the survival of the host in these harsh environments, by contributing to major nutrient cycles and host defensive strategies. Interestingly, the comparative genomics of sponge-associated bacterial isolates revealed that they were enriched in symbiotic lifestyle-related genes [145].

Additional studies applied a culture-dependent approach to characterize the cultivable bacterial communities and seldom test the biotechnological potential of bacterial isolates obtained from sponges [147,148,149,150,151]. Overall, the results reflected those obtained by culture-independent methods, with Gammaproteobacteria and Actinobacteria that predominated within the cultivable bacterial community, followed by Alphaproteobacteria, Bacteroidetes, and Firmicutes.

Antarctic benthic invertebrates, different from sponges, have been only rarely investigated for the associated bacterial community. Webster and Bourne [152] observed that cold-water coral-associated bacterial groups were dominated by Gammaproteobacteria across the soft coral *Alcyonium antarcticum*, with specific coral–microbial interactions across replicate coral samples within a site and between sites. Bacterial isolates were affiliated to the genera *Pseudomonas*, *Psychrobacter*, and *Shewanella* (Gammaproteobacteria); *Psychroserpens* and *Algoriphagus* (Bacteroidetes); and, finally, *Corynebacterium* (Actinobacteria). Among Cnidaria, the ice-dwelling anemone *Edswardsiella andrillae* lives beneath the Ross Ice Shelf with its body column in the ice and its mouth and tentacle crown in the water. A low–moderate level of diversity was observed, with Bacteroidetes and Proteobacteria represented by multiple lineages [153]. The authors found microbes that might help the animal survive, including endosymbiotic lineages (spanning from commensal to parasitic or pathogenic relationships) and others that could be Involved in chemical defense or elemental cycling.

González-Aravena et al. [154] analyzed the cultivable bacterial community in the coelomic fluid of the sea urchin *Sterechinus neumayeri* (Echinodermata), commonly observed in shallow subtidal zones around the Antarctic continent (up to 500 m depth). Isolates were mainly Gammaproteobacteria in the genera *Pseudoalteromonas*, *Psychrobacter*, *Shewanella*, and *Pseudomonas*, along with Flavobacteriaceae (among Bacteroidetes) and Actinobacteria. Recently, Schwob et al. [155] reported on the gut microbiota of the heart urchin *Abatus agassizii*. In contrast with González-Aravena et al. [154], the microbiota was characterized by a marked enrichment in Plantomycetacia, mostly represented by the *Blastopirellula* members. The authors individuated the genera *Desulfobacula* and *Spirochaeta* as potential keystone taxa, potentially host-selected.

Bacterial isolates from the oligochaete *Grania* sp. belonged to the genera *Flavobacterium*, *Pseudomonas*, *Psychrobacter*, and *Salinibacterium* [156]. The Antarctic ascidian *Synoicum adareanum* predominantly (97% of total sequences) harbored Proteobacteria (mainly Alpha- and Gammaproteobacteria), Bacteroidetes, and Verrucomicrobia [157]. Recently, further insights on the associated microbiome were reported by Murray et al. [158].

#### 4.3.2. Arctic Marine Environments

The microbial communities associated with Arctic benthic invertebrates have been less explored than the Antarctic ones. Two carnivorous sponge species, i.e., *Chondrocladia grandis* and *Cladorhiza oxeata*, were collected from Baffin Island (Canadian Arctic) and analyzed for the associated bacterial communities [159]. Consistentwith results achieved with Antarctic sponges, a high level of specificity was observed, with the two sponge species that hosted distinct communities. Interestingly, *C. grandis* showed certain bacterial taxa that were enriched in specific anatomical regions of the sponge body (e.g., within the root and root tip samples), suggesting the occurrence of species-specific core microbial assemblage involved in functional roles in carnivorous sponge metabolism or other biological processes. Associated bacteria were predominantly affiliates to either Proteobacteria (mainly Gamma- and Alphaproteobacteria) or Bacteroidetes (mainly Flavobacteria).

Sponge grounds (i.e., dense aggregations of large sponges) in Arctic-Boreal regions are often dominated by sponge species in the genus *Geodia*, which are characterized by a high-microbial abundance. *Geodia* generally co-occurs with the sponges *Stelletta* spp. and *Thenea* spp. [160]. Recently, Morganti et al. [161] reported on the prokaryotic community of the sponge species *G. parva*, collected across the peaks of extinct volcanic seamounts of the Arctic-Boreal Langseth Ridge. Chloroflexi markedly predominated over Acidobacteriota and Proteobacteria (Gamma- and Alphaproteobacteria). Chloroflexi and Acidobacteriota were significantly enriched in sponge tissues when compared to seawater. The authors also reported on the occurrence of microbial phyla that are classically known to be sponge symbionts (e.g., clades Entotheonellaeota, Nitrospirota, and Nitrospinota), along with a moderate abundance of Poribacteria and *Nitrosopumilus* (among Archaea).

The bacterial diversity was investigated in specimens of *Halichondria panicea* from Icelandic waters [162]. No marked differences were observed across different geographical locations. Alphaproteobacterial sequences were predominant, followed by Gammaproteobacteria, Flavobacteria, Planctomycetes, Cyanobacteria, and Verrucomicrobia. Interestingly, a strong decrease in the overall bacterial abundance was found within the sponge mesohyl after six months of transfer in a seawater aquarium. However, *‘Candidatus* Halichondribacter symbioticus’ remained the dominant bacterium, suggesting a certain stability of this sponge-bacterium association, which merits further investigation for studying sponge–symbiont co-evolution and functional interactions.

#### 4.3.3. Deep-Sea and Submarine Canyons

In recent years, extensive sequencing efforts have revealed enormous levels of microbial diversity in marine benthic invertebrates. However, most specimens are from shallow waters, thus limiting our current knowledge of invertebrate–microbe communities from the deep-sea.

The microbial biodiversity of deep-sea sponges has been seldom investigated, highlighting that the associated communities may represent an untapped source of potential microbial biodiversity [163]. By a cultivation approach, Brück and colleagues [164,165] discovered an *Entotheonella* species in *Discodermia dissoluta* from a depth of 150 m and later characterized the culturable anaerobes from *Geodia* sp. from depths of 200-350 m. *Lysobacter spongiicola* sp. nov. and *Pseudomonas pachastrellae* were isolated from the sponge *Pachastrella* sp. [166,167].

**Figure 3:**
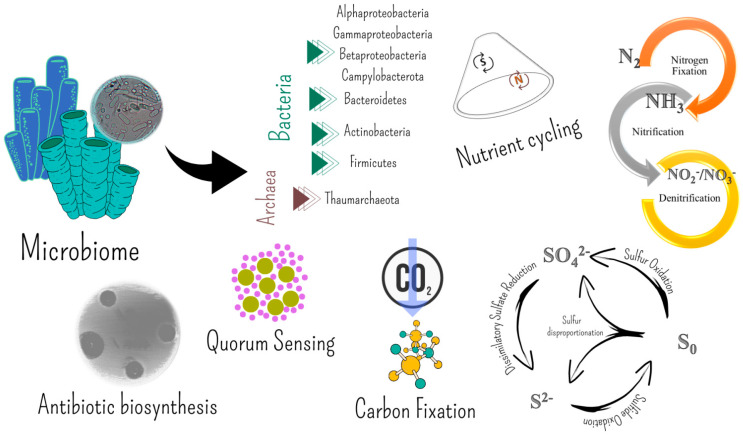
Main scientific evidences about bacterial taxa and functional processes reported for sponge microbiomes in extreme environments.

The application of advanced culture-independent techniques (e.g., next-generation sequencing) for the study of the prokaryotic communities associated with the the deep-sea sponges *Inflatella pellicula*, *Poecillastra compressa*, and *Stelletta normani* revealed that they included diverse bacteria and archaea, with *I. pellicula* in particular being dominated by Archaea, mainly ammonia-oxidizing Thaumarchaeota [26,168]. However, Bacteria were mainly represented by Proteobacteria, including Rickettsiales (Alphaproteobacteria), Desulfobacterales (Deltaproteobacteria), and Oceanospirillales and Chromatiales (Gammaproteobacteria). Members of the order Pirellulales (phylum Planctomycetes) were also abundant. Sulfur- and ammonia-oxidizing symbionts seem to be common also in non-carnivorous deep-sea sponges [29,31,111], implying that chemolithoautotrophy could be a widespread supplementary source of nutrition for deep-sea sponges in general [26] (Figure 3).

Betaproteobacteria and Campylobacterota, which were found in association with the sponge *Neamphius huxleyi*, were not found in seawater, despite the fact that Proteobacteria were the most prevalent bacteria in deep-sea water, thus highlighting the occurrence of sponge-specific lineages [169]. Furthermore, prokaryotes appeared to be strongly involved in nitrogen cycling (e.g., Proteobacteria, Thaumarchaeota and Actinomycetales) and CO_2_ fixation (e.g., Proteobacteria, Bacteroidetes, and Acidobacteria). More recently, the study of the prokaryotic diversity in the sponge species *Geodia barretti*, *Stryphnus fortis*, and *Weberella bursa*, was carried out on specimens collected along a depth gradient ranging from 244 to 1476 m [15]. The different water masses (across meso- and bathypelagic depths) had a major impact in the structuring of sponge-associated prokaryotic communities. In contrast with the compositional stability at phylum level, variations were observed at amplicon sequence variant (ASV) level, as evidence for a selective process for the required taxa from the environment.

Deep-sea coral reefs are often dominated by the colonial scleratinian corals *Lophelia pertusa* and *Madrepora oculata*, with the solitary *Desmophyllum cristagalli* that generally occurs. However, deep-sea coral-associated bacteria and their metabolisms still remain scarcely explored. The first records come back to 2006 [170,171]. Penn et al. [170] found that the bacterial community associated with octocorals (Bamboo coral, family Isididae, and Black coral at depths between 634 and 3,300 m) was dominated by Proteobacteria (mainly Alphaproteobacteria and Gammaproteobacteria), Firmicutes, Bacteroidetes, and Acidobacteria [170]. In the same year, Yakimov et al. [171] analyzed the active microbial communities associated with *L. pertusa* collected between 300 and 1000 m off the Cape of Santa Maria di Leuca (Apulian platform, Ionian Sea). The living coral harbored a specific microbial community, different from those observed for dead coral and sediment samples. *Holophaga-Acidobacterium* and *Nitrospira* divisions dominate the associated community, and more than 12% of all coral-associated riboclones formed a separate deep-branching cluster within the Alphaproteobacteria with no known close relatives. Since 2006, the microbial diversity of *L. pertusa* has been further deepened (e.g., [172,173,174,175]) and that of additional cold-water corals has been newly characterized, as in the case of *M. oculata* [176]; *Paragorgia arborea*, *Plumarella superba*, and *Cryogorgia koolsae* (among octocorals) [177]; and *Anthothela grandiflora*, *Anthothela* sp. and *Alcyonium grandiflorum* [178].

#### 4.3.4. Marine Caves

As far as we know, the only case of relationships between microorganisms and marine cave animals is reported by Vortsepnev et al. [179]. Microbial associations were found in three *Solenogastres* species, meiofaunal marine, shell-less, worm-like mollusks, from shallow Mediterranean marine caves. According to the authors, symbionts were strongly associated with the animal cuticle by obtaining energy or/and nutrients from the secretory cells in the epidermis.

Examples of benthic invertebrates from extreme marine environments analyzed to date for the symbiotic association with bacteria, as reported in the text above, are listed in Table 2.

## 5. Molecules Involved in the Microbe–Invertebrate Associations Become Biotechnologically Relevant

As reported in the introduction, host–microbe interactions are mediated by chemical compounds. By modifying their social connections or by directly controlling their immune systems, hosts can regulate their symbionts’ behavior. Thus, complex interactions between hosts and among associated bacterial populations may further stabilize coevolutionary patterns of symbiont communities [180]. The actual source of defensive molecules, for example, may be the organism itself or a microbial symbiont, or could derive by the interrelation between them. It is likely desirable for members of the associated microbial communities to be able to synthesize secondary metabolites that may improve the host natural chemical defense mechanisms. This is probably especially true for holobionts inhabiting extreme environments, which are exposed to extremes of pressure, salinity, and temperature. On the other hand, it remains often difficult to identify specific bacterial strains and chemical substances that are important for animal biology. Such difficulty lies in demonstrating that an identified substance is truly synthesized by a microorganism that was associated with the host. A bacterium isolated from an invertebrate is not always the relevant producer of a molecule in nature, even if it synthesizes the compound under laboratory conditions when in culture [181]. Fortunately, in some cases, evidence exists that some substances are both protective/useful for the host animal and produced by bacteria.

As stated by Lebar et al. [57], “*chemodiversity recapitulates biodiversity*”. Extreme ecosystems, with their rich biodiversity, could therefore be a potential source for bioactive molecules of therapeutic and biotechnological relevance. A number of studies have been addressed to extreme microbes isolated from abiotic matrices. On the other hand, works concerning the production of biomolecules by microorganisms associated with benthic invertebrates thriving in extreme environments are rarer, and the attention has been focused mainly to their envirotactic role, such as chemosynthesis.

**Figure 4:**
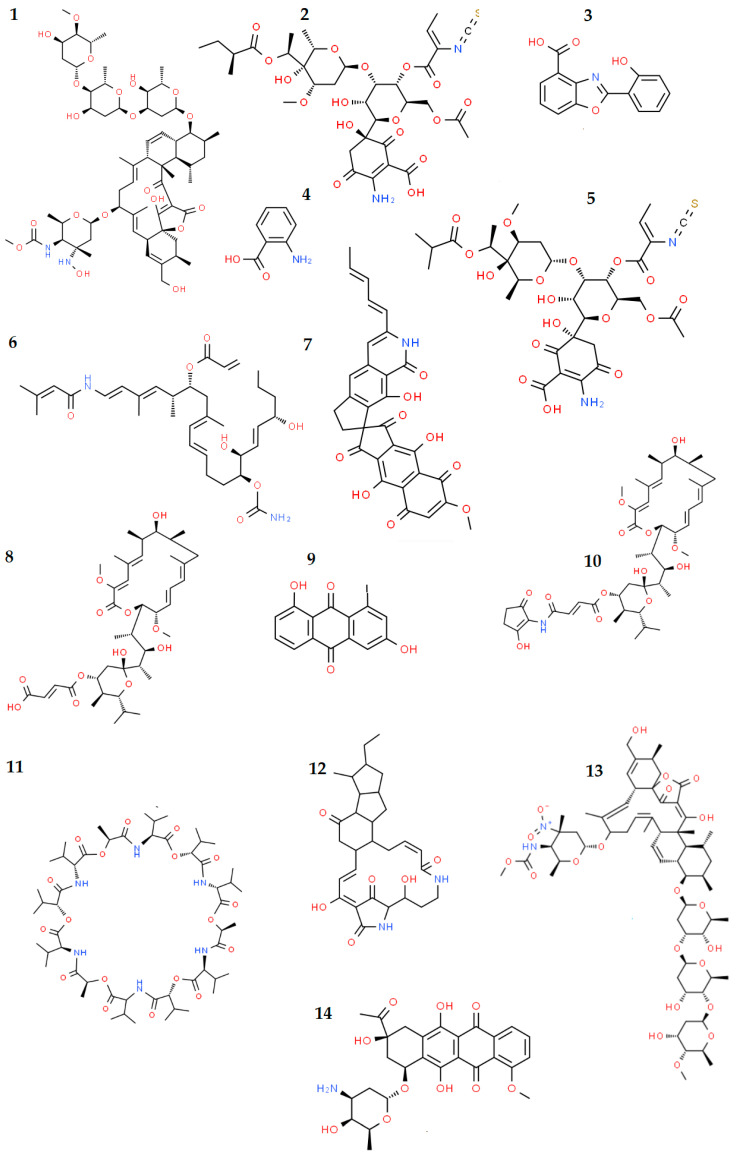
Chemical structures of molecules produced by bacteria associated with benthic invertebrates from extreme marine environments: **1.** lobophorin K; **2.** paulomycin A; **3.** caboxymycin; **4.** anthranilic acid; **5.** paulomycin B; **6.** palmerolide A; **7.** fredericamycin; **8.** bafilomycin C1; **9.** aloesaponarin II; **10.** bafilomycin B1; **11.** valinomycin; **12.** maltophilin; **13.** lobophorin B; **14.** daunomycin.

However, a number of biomolecules are known to be (often presumptively) involved in the interactions between bacteria and invertebrates. In the following paragraphs, the main achievements obtained in the search for bioactive molecules (or bioactivity) in invertebrate-associated bacteria from extreme environments are described. The chemical structures of the main bioactive molecules are shown in Figure 4.

### 5.1. Bioactive Molecules from Invertebrate-Associated Bacteria

Bioactive molecules are involved in the symbiotic association between benthic invertebrates and bacteria, including in extreme environments. A number of compounds, including those without a well-defined role in nature, could find applications in the fight against tumor cells or infective diseases. Nowadays, it is crucial to find new bioactive substances that are efficient against resistant microorganisms and safe to use in order to contrast the growing issue of antibiotic resistance, including the multi-drug resistance that exacerbates the situation [182]. Every year, infectious diseases pose a serious threat to human health, killing millions of people globally, particularly in underdeveloped countries. Current antibiotics, for example, are losing their ability to treat infections due to drug resistance, and organisms such as *Klebsiella pneumoniae*, *Pseudomonas aeruginosa*, and *Staphylococcus aureus* are once more a concern. This urgently requires novel medications [183]. Similarly, neoplastic growth urgently requires the search for novel and less toxic antitumor compounds with a broader antitumour spectrum [184].

The microbiome of marine benthic invertebrates is varied, and these bacterial populations create a wide range of bioactive compounds for resource competition and protection. The host microbiota is a potential source of clinically useful natural compounds as a result of these factors. A renaissance in the field is being fueled by recent developments in molecular, genetic, and analytical technologies that allow for the quick identification and characterization of new natural products with antimicrobial properties from microbes. Exploiting under-investigated and extreme ecological niches is one aspect of this discovery effort.

#### 5.1.1. Antibiotic and Antitumor Compounds

Polyketide synthase (PKS) and nonribosomal peptide synthase (NRPS) are involved in the alteration of a variety of substrates to create distinctive molecules with particular enzymatic, chemical, or antibacterial properties [185]. A distinct secondary metabolite is produced by each PKS or NRPS gene cluster, and the diversity of these gene clusters is a sign of the potential diversity of secondary metabolite products. The search for PKS and NRPS has been mainly reported for sponge-associated microbial communities, as follows.

The potential for secondary metabolite production of the microbiome of the deep- sea sponges *Inflatella pellicula*, *Poecillastra compressa*, and *Stelletta normani* (collected at 760–2900 m below sea level) was investigated by utilizing 454 pyrosequencing, targeting PKS and NRPS gene clusters [186]. The study, based on a culture-independent technique, revealed that a number of these genes occurred in the microbial communities, and they were mainly involved in the production of well-known types of bioactive substances, such as lipopeptides, glycopeptides, macrolides, and hepatotoxins. Most importantly, there was also a significant number of comparably unique sequences that are only loosely connected to the domains of well-known Type I PKS and NRPS sequences hosted in public databases, thus suggesting the production of still unknown antimicrobial compounds. Such an approach provided indirect means of exploring the secondary metabolism potential of sponge-associated communities as a source of novel small-molecules.

By a culture-dependent approach, Xin et al. [150] screened 46 Gram-positive isolates from the deep-sea sponges *R. nuda*, *R. racovitzae*, *M. mollis*, *R. antarctica*, and *H. balfourensis* for genes encoding PKS. PKS-I and PKS-II PCR products were detected in 70 and 85% of the isolates, respectively. A selection of 36 isolates were then used in subsequent bioassay analyses (antimicrobial tests) against *Erwinia carotovora*, *Xanthomonas campestris*, and *Xanthomonas oryzae*, with 32 of them inhibiting the growth of at least one test microorganism. Papaleo et al. [147], screening 140 bacterial isolates from three Antarctic sponge species (i.e., *Haliclonissa verrucosa*, *Anoxycalyx joubini* and *Lissodendoryx nobilis*) for the presence of *pks* genes, obtained an amplicon of the expected size (about 700 bp) only for *Pseudoalteromonas* strain TB41. The nucleotide sequence determined produced a significant match with sequences corresponding to proteins encoded by *pks* genes and associated with other *Pseudoalteromonas* isolates. Finally, PKS genes were also harbored by bacteria associated with the Antarctic tunicate *Synoicum adareanum* [157]. The authors suggested that they could encode palmerolide A (C_33_H_48_N_2_O_7_) (Figure 4), a macrocyclic polyketide displaying selective cytotoxicity toward melanoma by inhibiting V-ATPase, whose production had been previously ascribed to the host. Further analyses revealed that the metagenome-encoded biosynthetic machinery predicted to produce palmerolide A was found to be associated with the genome of a member of the *S. adareanum* core microbiome. Associated *Pseudovibrio* and *Microbulbifer* members were also shown to biosynthesize the macrolide palmerolide A [187].

Bacteria associated with sponge and corals from extreme marine environments have been proven to be prolific sources of bioactive molecules with antibacterial, antifungal, antiparasitic, and antiviral properties [188]. In some cases, only evidence of a certain bioactivity was reported, whereas the characterization of the molecule itself is not reported. With regard to sponges, the isolation of a new *Micromonospora* strain, designated 28ISP2-46T, from the microbiome of an unidentified Demosponge (mid-Atlantic deep-sea; 971 m depth) has been recently reported [189]. The whole-genome sequencing reveals the ability of 28ISP2-46T to synthesize a diverse array of natural products, with some of them exhibiting both antibiotic and antitumor properties (e.g., kosinostatin and isoquinocycline B). Both compounds, isolated from 28ISP2-46T fermentation broths, were shown to be effective against a diverse panel of pathogenic bacteria, including multidrug-resistant clinical isolates. The microbial communities associated with the Antarctic sponge *Isodictya setifera* included a *Pseudomonas aeruginosa* strain that was responsible for the production of a number of compounds, including phenazine alkaloid antibiotics, effective against Gram-positive microbes [190]. Mangano et al. [148] first reported on antagonistic interactions between 75 cultivable bacteria isolated from two Antarctic sponges, i.e., *Anoxycalyx joubini* and *Lissodendoryx nobilis*. A high percentage of active bacteria (81.3%) was determined, suggesting that microorganisms associated with sponges may be strongly competitive. According to the study, these kinds of interactions between bacteria associated with the same species of sponge and bacteria associated with different sponge species may be crucial in regulating and structuring the bacterial populations in their hosts. It is noteworthy that certain isolates also showed autoinhibition activity, which is typically dependent on the production of bacteriocins. These latter are polypeptides that kill bacteria from closely similar species and give the producing bacteria a selection advantage by partially limiting their own growth and living with competitors.

Further studies from Papaleo et al. [147] were focused on the effect against opportunistic pathogens associated with the *Burkholderia cepacia* complex (Bcc) that cause cystic fibrosis. A total of 140 bacterial strains were isolated from three Antarctic sponge species (i.e.,. *Haliclonissa verrucosa*, *A. joubini*, and *L. nobilis*) from shallow water of Terra Nova Bay. The sponge-associated bacteria were suggested as potential producers of volatile organic compounds (VOCs) that specifically inhibited *Burkholderia cepacia* without inhibiting other pathogenic bacteria [191]. The VOC activity resulted in being more effective in inhibiting the growth of Bcc bacteria than most of the commonly used antibiotics. VOCs are involved, as regulatory factors, in the interactions among microbes. Their synthesis was not induced by the presence of target strains. The volatile profiles of *Psychrobacter* isolates from *Anoxycalyx joubini* [147] were analyzed more in depth, with results highlighting that antimicrobial activity against Bcc bacteria might rely on a mixture of at least 30 VOCs, including sulfur-containing components (presumably responsible for the inhibition of Bcc strains) [192]. Sponge-associated isolates grown under variable growth conditions differently inhibited Bcc bacteria and presumably also produced non-volatile compounds, as suggested by the analysis of the core genome (which includes genes involved in the production polyketides, bacteriocins and siderophores) [193,194,195].

Actinobacterial isolates (49 strains) from different deep-sea sponge species (e.g., *Forcepia*, *Discodermia*, *Gorgonacea*, and *Leiodermatium*) were cultured and tested against a panel of bacterial pathogens considered as the most severe threats to human health: *Clostridium difficile*, *Pseudomonas aeruginosa*, methicillin-resistant *Staphylococcus aureus* (MRSA), and *Candida albicans* [183]. Isolates belonged to the well-known antibiotic-producing genus *Streptomyces*, as well as to rare Actinobacteria, such as *Actinomycetospora*, *Agrococcus*, *Leifsonia*, *Nocardiopsis*, *Promicromonospora*, *Rhodococcus*, *Salinispora*, and *Tsukamurella*. The crude extracts of 26 strains were active, with the majority of them (21) inhibiting the growth of MRSA. Among those showing antifungal activity, *Streptomyces* strains R786 and R818 (both from the sponge *Forcepia* sp.) were particularly effective. *Streptomyces* strain N217 (from the sponge *Forcepia* sp.) showed a broad antibiotic activity (probably dependent on the production of different types of compounds), except against *C. difficile*. Three strains (*Streptomyces* spp. N201, N203, and N217; all from *Forcepia* sp.) exhibited anti-*Pseudomonas* activity. Only *Salinispora* strain M864 (cultivated from a sponge of the family Oceanapiidae collected from Bahamas) was able to produce metabolites active against *C. difficile*. Further analyses on the metabolites produced by M864 were addressed to test their cytotoxicity and therapeutic potential, by the MTT cell viability assay performed using HepG2 (human liver carcinoma) and HEK (Human embryonic kidney) cell lines. Results revealed that M864 metabolites likely contain natural products which are more potent than vancomycin against *C. difficile*, and the authors deserved further investigation. The addition of the rare Earth salt lanthanum chloride (LaCl_3_) to the culture medium was shown to be an effective elicitor through the activation of cryptic gene clusters, thus highly enhancing opportunities to discover novel natural products. Antifungal or antibacterial activities in strains that were inactive under normal cultivation conditions were induced by LaCl_3_.

With respect to corals, Braña et al. [196] reported on the characterization of a new natural product, lobophorin K (C_61_H_92_N_2_O_20_), obtained from the *Streptomyces* sp. M-207, previously isolated from the cold-water coral *Lophelia pertusa* collected at 1800 m depth in a submarine canyon [197] (Figure 4). The chemical analysis revealed that the extract obtained from fermentation broths of strain M-207 contained also already known lobophorins A and B. Lobophorin K showed cytotoxic activities against two human tumor cell lines (i.e., pancreatic carcinoma and breast adenocarcinoma) and a moderate antibiotic activity against pathogenic Gram-positive bacteria (i.e., *Staphylococcus aureus*). Sarmiento-Vizcaíno et al. [198] deeply characterized the actinobacterium *Myceligenerans cantabricum* sp. nov. (strain M-201T), isolated from a deep-sea solitary scleractinian disk coral (Fam. Caryophillidae) collected at 1500 m depth in the Avilés Canyon in the Cantabrian Sea (Asturias, Spain). The isolate showed antibiotic activity against *Escherichia coli*, *Micrococcus luteus*, and *Saccharomyces cerevisiae*. The same authors [199] obtained different *Streptomyces* isolates from different marine and terrestrial environments, including organisms from deep-sea ecosystems (e.g., gorgonian and solitary corals and other invertebrates, such as Annelida, Echinodermata, Arthropoda, and Porifera) living up to 4700 m depths and at a temperature of 2–4 °C in the submarine Avilés Canyon. *Streptomyces* isolates produced an array of bioactive compounds with diverse antibiotic and cytotoxic activities, including paulomycins A (C_34_H_46_N_2_O_17_S) and B (C_33_H_44_N_2_O_17_S) (with antibacterial activity), maltophilins with antifungal and cytotoxic properties (e.g., antimycins and 6-epialteramides), and the fredericamycin (C_30_H_21_NO_9_; an antitumor compound) (Figure 4).

A total of 16 Actinobacterial strains in the genera *Streptomyces*, *Myceligenerans*, and *Micromonospora* were isolated from corals and other benthic organisms (e.g., Porifera, Annelida, Echinodermata, Arthropoda) at depths ranging from 1500 to 4700 m [200]. They were screened for antibiotic activity against a panel of important resistant clinical pathogens, including Gram-positive and Gram-negative bacteria and the yeast *Saccharomyces cerevisiae*. *Streptomyces* M-231, isolated at 4700 m in association to the decapod *Colossendeis colossea*, was particularly active. Complex metabolic profiles were obtained for most extracts. The identified products mainly possess antibacterial (e.g., paulomycins A and B, caboxamycin (C_14_H_9_NO_4_), aloesaponarin II (C_15_H_10_O_4_), anthranilic acid (C_7_H_7_NO_2_)), antifungal (bafilomycins B1 (C_44_H_65_NO_13_) and C1 (C_39_H_60_O_12_), maltophilin (C_29_H_38_N_2_O_6_)), antitumor (caboxamycin, daunomycin (C_27_H_29_NO_10_), galtamycin), antiparasitic (paulomycins A and B, valinomycin (C_54_H_90_N_6_O_18_)), antiviral (valinomycin), and anti-inflammatory (lobophorin B, C_61_H_90_N_2_O_21_) activities (Figure 4). Most extracts were found to be moderately active against both HeLa (from cervical carcinoma) and HCT116 (from colorectal carcinoma) cell lines. Highly diluted (1:100) extracts from *Streptomyces* strains M-157 (associated to a stony coral; (2000 m depth) and M-192 (from an actinia; 4700 m depth) maintained their strong activity.

Similarly, a total of 111 isolates obtained from different benthic individuals of shallow hydrothermal system in Eyjafjörður (northern Iceland) showed antimicrobial activity with different antimicrobial patterns [131]. Specifically, a promising antimicrobial activity against *Staphylococcus aureus, Candida albicans*, and *Escherichia coli* was exhibited by bacterial symbionts, mainly Actinobacteria members, of anemones, ascidians, macroalgae, sponges, and one nudibranch.

#### 5.1.2. Antibiofilm Capabilities and Quorum Sensing

Microbial populations immersed in an extracellular polymeric matrix are known as microbial biofilms. As “protective clothing,” biofilms can shield microorganisms from ultraviolet radiation, extreme temperatures and pH, high salinity, high pressure, deficiency in nutrients, and antibiotics [201]. Thus, biofilm enhances the survival and metabolism of microbes under adverse conditions. Both animated and inanimate surfaces immersed in seawater can rapidly become coated with a biofilm. This happens because surfaces are often enriched in organic material, as a consequence of physical (e.g., the adsorption of molecules) and biological (e.g., secretion of mucus into the surrounding environment) processes [202,203].

The processes of bacterial adhesion and biofilm formation are crucial for both environmental and human health. Biofilm has a significant impact on the marine industries, where it enhances biofouling (the accumulation of macrofoulers on the surface of human-made underwater objects) and speeds up corrosion [204]. From an ecological perspective, several marine organisms synthetize antifouling compounds to avoid the settlement of undesirable encrusting organisms on their surface. Antifouling activity in extreme environments has been reported for compounds produced, for example, by Cnidaria (i.e., *Alcyonium paessleri* and *Gersemia antarctica*) [205] and other benthic Antarctic invertebrates, including Porifera and Bryozoa [206,207]. To date, no studies have specifically addressed the production of antifouling molecules by bacteria associated with benthic invertebrates from extreme environments, even if it is not to be excluded a major role played in the process by the bioactive molecules reported in the paragraph above.

In many industrial fields, including the food, medical, and paper industries, biofilm is a significant issue [208,209]. Biofilm-forming pathogens are frequently responsible for bacterial infections in humans and often linked to persistent infections, including those that cause cystic fibrosis, urethritis, otitis, periodontitis, and endocarditis [210]. By shielding bacteria from the host immune system and making them less susceptible to antimicrobial treatments, the biofilm lifestyle promotes the development of persistent infections, which are notoriously difficult to treat [208]. Recently, Antarctic-sponge associated bacteria were assayed for the production of antibiofilm compounds against two clinically relevant microorganisms, i.e., *Pseudomonas aeruginosa* and *Staphylococcus aureus*. Tested strains belonged to the genera *Colwellia*, *Pseudoalteromonas*, *Shewanella*, and *Winogradskyella* and were derived from different sponges species (i.e., *Hemigellius pilosus*, *Haliclona dancoi*, *Tedania charcoti*, *Haliclona virens*, *Anoxycalyx joubini*, *Calyx arcuarius*, *Haliclonissa verrucosa*). The results suggested that cell-free supernatants of cold-adapted bacterial isolates might contain antibiofilm compounds with surfactant properties, interfering with the initial adhesion by *P. aeruginosa* and *S. aureus* on surfaces. The study confirmed the sponge bacterial symbionts as a potential source of molecules useful in contrasting bacterial infection diffusion. However, the molecule characterization was not reported [211].

Biofilm formation and development is seldom influenced and controlled by quorum sensing (QS), an intercellular signaling system in bacterial populations. Two well-known systems are based on acyl-homoserine lactone (AHL) and autoinducing peptide (AIP) synthesis by Gram-negative and Gram-positive species, respectively, along with the autoinducer-2 (AI-2) QS system in both Gram-negative and -positive bacteria [212]. Engineered QS systems of bacterial origin could find several applications in biotechnology, e.g., in the production of biochemicals, tissue engineering, and mixed-species fermentations, building QS-based microbial biosensors and QS-based biocontrol strategies, as well as in a viable strategy for the reduction in biofouling (based on QS inhibition) [213]. To date, evidence of QS phenomenon in extremophilic bacteria associated with benthic invertebrates exist only for Antarctic Porifera in the species *A. joubini*, *L. nobilis*, and *Myxodorys hanitschi* [214]. The authors examined 211 Gram-negative bacterial strains for the production of AHLs using three different AHL biodetection systems (*Agrobacterium tumefaciens* pZLR4, *Chromobacterium violaceum* CV026, and *Pseudomonas putida* pKR-C12). Isolates which were able to activate at least one of the monitor systems belonged to bacterial genera that are involved in surface colonization by biofilm production.

### 5.2. Extracellular Polymeric Substances

Extracellular polymeric substances (EPSs) of microbial origin assume several ecological roles in the natural environments. Bacterial cell aggregation, flocculation and biofilm formation, cell recognition, adhesion to surfaces, water retention to prevent desiccation, and the ability to serve as a protective barrier are all mediated by extracellular polymeric substances. In cold environments, where freeze–thaw cycles commonly occur, EPSs are produced by bacteria to front cryoinjuries derived from low temperatures, i.e., the formation of ice-crystals causing cell damage. Recently, Decho and Gutierrez [215] reviewed major physical and chemical properties and functions in the marine environment.

EPSs find a plethora of biotechnological applications mainly falling into the pharmaceutical field as therapeutic agents and in several industrial contexts, i.e., as stabilizers and additives in food production, emulsifiers in cosmetic production, andanti-freezing compounds [216,217,218,219].

To date the production of EPSs by invertebrate-associated bacteria from extreme marine environments has been reported mainly for marine hydrothermal systems and Antarctica, as it is reported in the following paragraphs.

#### 5.2.1. EPS from Deep-Sea Hydrothermal Vents

A mucoid substance secreted by tiny glands lining the dorsal intersegmental space of the deep-sea worm *Alvinella pompejana* feeds the associated bacteria, which in turn form a protective “fleece-like” covering. It is supposed that symbiotic bacteria could detoxify the water within the worm’s tube from toxic chemicals (e.g., sulfides and heavy metals) [104]. The EPS-producing *Alteromonas macleodii* subsp. *fijiensis* biovar deepsane (strain HYD657; among Gammaproteobacteria) was isolated from the epidermis of an *A. pompejana* specimen. The high-molecular-weight (1.6 × 10^6^ Da) biopolymer produced by the strain HYD657, called Deepsane^TM^, possesses intriguing biological activities, and it is the first marine exopolysaccharide to be commercialized for cosmetics [220,221]. Its commercial name is Abyssine^®^ (patent PCT 94907582-4), and it is used to soothe and reduce irritation of sensitive skin against chemical, mechanical, and UVB aggressions [222]. The gross chemical composition revealed the absence of hexosamines nor sulfate in the polymer, while proteins were found to be below 0.5%. Neutral and acidic sugars accounted for 58 and 30% of the total sugars, respectively. The monosaccharide composition includes glucose, galactose, rhamnose, fucose, and mannose as neutral sugars, along with glucuronic and galacturonic acids. The HYD657 EPS also contained an unusual sugar (a diacidic hexose), identified as 3-0-(1 carboxyethyl)-d-glucuronic acid (also found in another EPS secreted by an *Alteromonas* sp. from deep-sea vents [223]).

*A. pompejana* is a prolific source of EPS-producing bacteria. Another *Alteromonas macleodii* isolate, i.e., strain HYD-1545, was obtained from *A. pompejana* by Vincent et al. [224]. Galactose, glucose, glucuronic acid, galacturonic acid, and 4,6-O-(1-car-boxyethylidene)-galactose (GalX; X as pyruvate) were major components of the polymer produced by HYD-1445 (molar ratio 2.5:3:2:2:1), commonly retrieved in acidic polysaccharides of marine origin. Uronic acids accounted for 40% of the EPS crude extract. A bacterial-mediated metal detoxification, due to EPS production, has been suggested for *A. pompejana* specimens exposed to high concentrations of toxic chemicals. Finally, *Vibrio diabolicus* strain HE800 is an EPS-producing facultative anaerobe isolated from the dorsal integument of *A. pompejana* [225]. Differently from *Alteromonas* HYD657, large proportions of uronic acids and hexosamines (by equal amounts) were determined by colorimetric assays in the EPS, designated HE800 [226,227]. Glucuronic and galacturonic acids were detected by gas chromatography, as well as amino sugars such as glucosamine and galactosamine. The C-13 nuclear magnetic resonance spectrum showed that it consists of a linear tetrasaccharide repeating unit with two N-acetyl-hexosamine and two glucuronic acid residues. Other signals were related to the methyl of acetyl groups, suggesting the occurrence of N-acetylation of the hexosamines. No sulfate group was present. HE800 EPS (patent US 7015206B2) possesses a high biotechnological value, especially for human health. It has been proposed as a component of medicinal products due to its cicatrizing activity and bone regeneration [228]. Fast bone healing is also efficiently induced by HE800 thanks to its capacity to bind calcium. Goudenège et al. [229] sequenced and analyzed the genome of *V. diabolicus* HE800 and individuated a gene cluster (i.e., the *syp* cluster) that can be assigned to EPS biosynthesis. These results will contribute to elucidating the EPS biosynthetic machinery functioning, including the regulatory network, thus possibly helping in improving the production yield.

The strain GY785, identified as *Alteromonas infernus*, was isolated from fluids collected among a dense population of the giant worm *Riftia pachyptila* in the proximity of an active hydrothermal vent [230]. In the presence of glucose, *A. infernus* produced two distinct EPSs, one (EPS-1) being soluble recovered from the supernatant and a second one (EPS-2) rich in proteins (40%) and not chemically linked to the polysaccharide core, forming a gelatinous matrix with bacterial cells. EPS-1 (molecular weight 1x10^6^ Da) was an acidic heteropolysaccharide containing galactose, glucose, galacturonic, and glucuronic acids (molar ratio 1:1:0.7:0.4). Both the carbohydrate composition (primarily in terms of monosaccharide ratios) and sulfate content (10%) of EPS GY785 slightly differed from EPS excreted by other bacterial isolates from deep-sea hydrothermal vents. Uronic acids accounted for up to 42%, while hexosamines were in trace amounts (0.7%). The structure of the EPS GY785, elucidated by Roger et al. [231], resulted in repeating units of a monosaccharide. Due to the negative charge conferred to the EPS by the uronic acids, the authors suggested that it could be applied in wastewater treatment or in the recovery of metals. Conversely, its low viscosity makes EPS GY785 not suitable for applications in the food industry as gelling or emulsifying agents. Further insights highlighted that several oversulfated low-molecular-weight EPS fractions, with uronic acid and sulfate contents, possessed anticoagulant and antithrombotic activity, similarly to heparin [232]. Recently, Heymann et al. [233] reported on the effective inhibition of both the migration and invasiveness of osteosarcoma cells in vitro, as well as the establishment of lung metastases in vivo, by oversulfated low-molecular-weight EPS fractions. Finally, the EPS GY785 also stimulates the chondrogenesis of mesenchymal stem cells, which are an attractive source of cells for cartilage regeneration [234].

#### 5.2.2. EPSs from Cold Marine Systems

To date, EPS-producing bacteria (genera *Colwellia*, *Shewanella*, and *Winogradskyella*) have only been isolated from Antarctic sponges (i.e., *Tedania charcoti*, *Haliclonissa verrucosa*, and *Hemigellius pilosus*) from Terra Nova Bay [235]. EPS production was higher at sub-optimal incubation temperature (4 °C) and cell viability after four subsequent freeze–thaw cycles were better sustained in the presence of EPSs. This finding suggests the cryoprotective role played by these EPSs under low-temperature stress conditions. Chemically, the EPSs showed a moderate carbohydrate content (range 15–28%), and the presence of proteins (range 3–24%) and uronic acids (range 3.2–11.9%). EPSs mainly differed in the relative proportion of main sugars. EPS from *Shewanella* sp. CAL606 showed glucose, galactose, mannose, galactosamine, glucuronic acid, and galacturonic acid (molar ratio 1:1:0.9:0.6:0.3:0.1) as main sugars. *Colwellia* sp. GW185 produced an EPS composed of glucose, mannose, galactose, galactosamine, glucuronic acid, and galacturonic acid (molar ratio 1:1:0.7:0.7:0.3:0.04). Finally, the EPS produced by *Winogradskyella* sp. CAL396 contained mannose, arabinose, galacturonic acid, glucuronic acid, galactose, glucose, and glucosamine in the relative proportions 1:0.9:0.4:0.3:0.2:0.2:0.01. Finally, the main sugars identified in the EPS from *Winogradskyella* CAL384 were glucose, mannose, galacturonic acid, arabinose, galactose, glucosamine, and glucuronic acid (relative proportions 1:0.5:0.3:0.25:0.1:0.1:0.1).

The excellent emulsifying activity toward hydrocarbons (i.e., hexane, octane, hexadecane, and tetradecane), with a stable emulsion index (E24) higher than those measured for synthetic surfactants (i.e., Tween 80 and Triton-X), was most likely caused by the high protein content in the EPSs produced by *Winogradskyella* sp. CAL384. The strains tolerated higher levels of mercury and cadmium (up to 10000 ppm) in the presence of EPS, suggesting a detoxifying role. This capability was probably reliant on the presence of uronic acids and sulfate groups, which can function as ligands for cations in the EPS molecules.

The main achievements on the bioactivity of invertebrate-associated bacterial isolates and communities from extreme marine environments are summarized in Table 3.

## 6. Conclusions

To date, marine extreme habitats have not been properly exploited as a resource in the framework of chemodiversity-discovery efforts. The study of molecules involved in the invertebrates–microbes interactions under extreme environmental conditions is unquestionably still in its infancy. Symbiotic associations certainly warrant further exploration, even if high costs are needed and technical challenges exist for sampling at remote and extreme areas of the globe.

This review makes evident that several extreme environments (i.e., hypersaline environments, submarine caves) are almost totally unexplored or underexplored from both ecological (e.g., microbial biodiversity) and biotechnological points of view. On the other hand, based on available data, our current knowlegde appears highly fragmented and probably relies on non-systematic sampling and research activities. Most of the achievements on the symbiotic relationships often pause to outline the diversity profile of the symbiotic bacterial communities. We are still very far from defining what environmental conditions (including the interaction between bacteria and their hosts) stimulate the production of the metabolites involved in the bacteria–host relationship, whether different substances are produced in case of variations in the surrounding environment, and what exactly is the role these molecules play. Although the role of the bioactive molecules is recognized in establishing and maintaining association relationships between microorganisms and higher animals, it is difficult to pinpoint the exact role of molecules in the relationship itself. It could be linked to the mechanisms of cellular communication and signaling, or to the role of regulating cell proliferation for maintaining population balance, or to the defensive strategy against potential pathogens, or finally to a set of all these functions.

Further efforts are needed to elucidate the chemical structures of the purified molecules and their exact functioning mechanism. It can be presumed that the true biotechnological potential of our “extreme” oceans has not yet been fully evaluated and that we have merely “scratched” the surface. Overall, the main achievements reviewed here suggest that the marine production of natural products may be more widespread than previously supposed and demonstrate the value of targeting the microbiome of benthic invertebrates from extreme environments as a source of novel microbial life with exploitable biosynthetic potential.

## Figures and Tables

**Table 1:** Main features of extreme marine environments.

Extreme Environment	Main Extreme Features *(Alone or in Combination)*	Stable, Unstable, or Temporary
Deep-sea	High pressure; low temperature; scarcity of food	Stable
Deep-sea hydrothermal vents	High temperatures; low O_2_ level; absence of light; presence of sulfide and heavy metals; high pressure	Temporary
Shallow hydrothermal vents	High temperatures; low O_2_ level; presence of sulfide and heavy metals	Temporary
Cold seeps	Low temperatures; high levels of sulfide, methane, and bicarbonate; high pressure; low O_2_ level	Temporary
Cold waters	Low and stable temperature; seasonal variations in light intensity and primary production; variations in salinity	Stable
Submarine canyons	Instability and physical disturbance; turbidity currents; low temperatures; high pressure; absence of light	Unstable
Hypersaline environments	High salinity; high UV irradiance; low nutrient availability; low O_2_ level	Unstable
Submarine caves	Darkness; low nutrient availability; limited accessibility; salinity gradient; presence of sulfides; deoxygenation	Stable

**Table 2:** Examples of benthic invertebrates analyzed to date for the symbiotic association with bacteria.

Extreme Environment	Invertebrate Host	Phylum or Sub-phylum	Main Associated Bacteria	Bacterial Function(s) in the Symbiosis	References
DSHVs	*Alviniconcha* sp.	Mollusca	Gammaproteobacteria Campylobacterota	Sulfur cycle	[12]
	*Alvinella pompejana* Desbruyeres & Laubier, 1980	Annelida	Gammaproteobacteria Campylobacterota	- Detoxification of sulfide and heavy metals - Sulfur oxidation	[98,99,100,101,103]
	*Bathymodiolus azoricus* Cosel & Comtet, 1999	Mollusca	Gammaproteobacteria	Sulfur and methane oxidation	[110]
	*Bathymodiolus puteoserpentis* Cosel, Métivier & Hashimoto, 1994	Mollusca	Gammaproteobacteria	Sulfur and methane oxidation	[110]
	*Helicoradomenia* sp.	Mollusca	Alphaproteobacteria Gammaproteobacteria	Not reported	[109]
	*Kiwa* spp.	Crustacea	Gammaproteobacteria Campylobacterota	-Sulfur oxidation - Detoxification	[96]
	*Lamellibrachia anaximandri* Southward, Andersen & Hourdez, 2011	Annelida	Gammaproteobacteria	Sulfur oxidation	[112]
Cold-seeps	*Escarpia* sp.	Annelida	Planctomycetes Proteobacteria	Ammonia oxidation	[116]
	*Hymedesmia (Stylopus) methanophila* Cárdenas, 2019	Porifera	Gammaproteobacteria	-Sulfur and methane oxidation -Hydrocarbon degradation	[30]
	*Iophon methanophila* Cárdenas, 2019	Porifera	Gammaproteobacteria	-Sulfur and methane oxidation -Hydrocarbon degradation	[30]
	*Lamellibrachia* spp.	Annelida	Gammaproteobacteria	Sulfur oxidation	[110]
SHVs	*Actinia equina* (Linnaeus, 1758)	Cnidaria	Gammaproteobacteria Campylobacterota	Sulfur oxidation	[130]
	*Anemonia viridis* (Forsskål, 1775)	Cnidaria	Gammaproteobacteria	Sulfur oxidation	[132,133]
	*Astroides calycularis* (Pallas, 1766)	Cnidaria	Bdellovibrionaceae Verrucomicrobia	-Nitrogen fixation -Degradation of organic nitrogen	[134]
	*Hymeniacidon* sp.	Porifera	Alphaproteobacteria Gammaproteobacteria	Not reported	[130]
	*Xenograpsus testudinatus* Ng, Huang & Ho, 2000	Crustacea	Gammaproteobacteria Campylobacterota	-Sulfur cycling -Detoxification	[126]
Deep-sea	*Characella* sp.	Porifera	Gammaproteobacteria	Sulfur oxidation	[110]
	*Discodermia dissolute* Schmidt, 1880	Porifera	Entotheonellaeota	Not reported	[164,165]
	*Geodia* sp.	Porifera	Chloroflexi Acidobacteria Gammaproteobacteria Alphaproteobacteria	Not reported	[166,167]
	*Inflatella pellicula* Schmidt, 1875	Porifera	Gammaproteobacteria Alphaproteobacteria Deltaproteobacteria Planctomycetes	Sulfur oxidation	[26,168]
	*Lophelia pertusa* (Linnaeus, 1758)	Cnidaria	Alphaproteobacteria Gammaproteobacteria Firmicutes Bacteroidetes Acidobacteria	Not reported	[170,171,172,173,174,175]
	*Neamphius huxleyi* (Sollas, 1888)	Porifera	Betaproteobacteria Campylobacterota	-Nitrogen cycling -CO_2_ fixation	[169]
	*Poecillastra compressa* (Bowerbank, 1866)	Porifera	Gammaproteobacteria Alphaproteobacteria Deltaproteobacteria Planctomycetes	Sulfur oxidation	[26,163]
	*Stelletta normani* Sollas, 1880	Porifera	Gammaproteobacteria Alphaproteobacteria Deltaproteobacteria Planctomycetes	Sulfur oxidation	[26,163]
Antarctic seawater	*Abatus agassizii* Mortensen, 1910	Echinodermata	Gammaproteobacteria Bacteoidetes Actinobacteria	Sulfur cycling	[155]
	*Alcyonium antacticum* Wright & Studer, 1889	Cnidaria	Gammaproteobacteria Bacteroidetes Actinobacteria	Not reported	[152]
	*Anoxycalyx (Scolymastra) joubini* (Topsent, 1916)	Porifera	Gammaproteobacteria Bacteroidetes Actinobacteria	Not reported	[147,148,151]
	*Grania* sp.	Annelida	Gammaproteobacteria	Not reported	[156]
	*Haliclonissa verrucosa* Burton, 1932	Porifera	Actinobacteria Gammaproteobacteria	Not reported	[147,151]
	*Hemigellius pilosus* (Kirkpatrick, 1907)	Porifera	Gammaproteobacteria Bacteroidetes Actinobacteria	Not reported	[141,142,146,148,151]
	*Hymeniacidon torquata* Topsent, 1916	Porifera	Alphaproteobacteria Gammaproteobacteria	Not reported	[136,139,140,145]
	*Isodictya bentarti* Rios, Cristobo & Urgorri, 2004	Porifera	Gammaproteobacteria	Not reported	[137]
	*Lissodendoryx nobilis* (Topsent, 1916)	Porifera	Gammaproteobacteria Bacteroidetes Actinobacteria	Not reported	[147,148,151]
	*Mycale (Oxymycale) acerata* Kirkpatrick, 1907	Porifera	Gammaproteobacteria Bacteroidetes Actinobacteria	Not reported	[135,138,139,141,142,143,145]
	*Sterechinus neumayeri* (Meissner, 1900)	Echinodermata	Gammaproteobacteria Bacteroidetes	Not reported	[154]
	*Tedania charcoti* Topsent, 1907	Porifera	Gammaproteobacteria Bacteroidetes Actinobacteria	Not reported	[151]
	*Edswardsiella andrillae* Daly, Rack & Zook, 2013	Cnidaria	Proteobacteria Bacteroidetes	Not reported	[153]
	*Synoicum adareanum* (Herdman, 1902)	Chordata	Alphaproteobacteria Gammaproteobacteria Bacteroidetes Verrucomicrobia	Not reported	[157,158]
Arctic seawater	*Halichondria panicea* (Pallas, 1766)	Porifera	Alphaproteobacteria Gammaproteobacteria Bacteroidetes Planctomycetes Cyanobacteria Verrucomicrobia	Not reported	[162]
	*Chondrocladia grandis* (Verrill, 1879)	Porifera	Alphaproteobacteria Gammaproteobacteria Bacteroidetes	Not reported	[159]
	*Cladorhiza oxeata* Lundbeck, 1905	Porifera	Alphaproteobacteria Gammaproteobacteria Bacteroidetes	Not reported	[159]

**Table 3:** Main achievements on invertebrate-associated bacterial isolates and communities from extreme marine environments showing bioactivity. Details are given in the text above.

Invertebrate Host(s), Phylum or Subphylum	Bacterial Genus/Species/Strain *	Biomolecule(s) or (Potential) Bioactivity **	Reference(s)
Deep-sea hydrothermal vents			
*Alvinella pompejana*, Annelida	*Alteromonas macleodii* subsp. *fijiensis* biovar deepsane (strain HYD657)	High-molecular weight biopolymer Deepsane^TM^ with application in cosmetics	[220,221,222]
*Alvinella pompejana*, Annelida	*Alteromonas macleodii* strain HYD-1545	Possible application of produced EPSs in the detoxification of metal-contaminated environments	[224]
*Alvinella pompejana*, Annelida	*Vibrio diabolicus* strain HE80	Possible application of produced EPSs as a component of medicinal products due to its cicatrizing activity and bone regeneration	[225,226,227,228]
*Riftia pachyptila*, Annelida	*Alteromonas infernus* strain GY785	Possible application of produced EPSs in wastewater treatment or in the recovery of metals; oversulfated low-molecular-weight EPS fractions showed: anticoagulant, antithrombotic and antitumor activities; possible application in cartilage regeneration	[225,232,233,234]
Shallow hydrothermal vents			
Several invertebrates in the phyla Cnidaria, Tunicata, Porifera and Mollusca	Mainly Actinobacteria isolates	Antibiotic activity against pathogenic bacteria	[131]
Deep-sea and submarine canyons			
*Homaxinella balfourensis*, *Myxilla mollis*, *Radiella antarctica*, *Rossella nuda*, *Rossella racovitzae*, Porifera	Several strains	Occurrence of genes coding for PKS	[150]
Unidentified Demosponge, Porifera	*Micromonospora* strain 28ISP2-46T	Production of antibiotic and antitumor compounds (e.g., kosinostatin and isoquinocycline B)	[189]
*Inflatella pellicula*, *Poecillastra compressa, Stelletta normani,* Porifera	Whole bacterial communities	Potential production of lipopeptides, glycopeptides, macrolides, and hepatotoxins	[163]
*Forcepia*, *Discodermia*, *Gorgonacea*, *Leiodermatium*, Porifera	Actinobacterial isolates; mainly *Streptomyces* strains	Antibiotic activity against MRSA and *Candida albicans*	[183]
Unidentified sponge (Fam. Oceanapiidae), Porifera	*Salinispora* strain M864	Antibiotic activity against *Clostridium difficile*; antitumor activity	[183]
*Lophelia pertusa,* Cnidaria	*Streptomyces* sp. M-207	Production of lobophorin K with antitumor and antibiotic activities	[196,197]
Unidentified coral (Fam. Caryophillidae), Cnidaria	*Myceligenerans cantabricum* strain M-201T	Antibiotic activity against pathogenic bacteria	[198]
Several invertebrates in the phyla Annelida, Echinodermata, Arthropoda, and Porifera	*Streptomyces* isolates	Production of compounds with antibiotic and/or cytotoxic activities (e.g., paulomycins A and B, maltophilins, antimycins, 6-epialteramides, fredericamycin)	[199]
Several invertebrates in the phyla Annelida, Echinodermata, Arthropoda, Porifera, Cnidaria	Actinobacteria isolates (genera *Streptomyces*, *Myceligenerans*, *Micromonospora*)	Antibiotic activity against a panel of resistant clinical pathogens	[197]
*Colossendeis colossea*, Arthropoda	*Streptomyces* strains M-231, M-157, M-192	Production of compounds with antibacterial (e.g., paulomycins A and B, caboxamycin, aloesaponarin II, anthranilic acid), antifungal (bafilomycins B1 and C1, maltophilin), antitumor (caboxamycin, daunomycin, galtamycin), antiparasitic (paulomycins A and B, valinomycin), antiviral (valinomycin), and anti-inflammatory (lobophorin B) activities.	[197]
Antarctic waters			
*Anoxycalyx joubini, Haliclonissa verrucosa*, *Lissodendoryx nobilis*, Porifera	Several strains; *Pseudoalteromonas* sp. TB41	Potential production of antibiotic compounds against Bcc pathogens; occurrence of genes coding for PKS	[192]
*Isodictya setifera*, Porifera	*Pseudomonas aeruginosa*	Antibiotic compounds (phenazine alkaloid antibiotics)	[190]
*Synoicum adareanum*, Tunicata	*Pseudovibrio* and *Microbulbifer* strains	Antitumor compounds (e.g., palmerolide A); occurrence of genes coding for PKS	[157,187]
*Hemigellius pilosus, Haliclona dancoi, Tedania charcoti, Haliclona virens, Anoxycalyx joubini, Calyx arcuarius, Haliclonissa verrucosa,* Porifera	Several strains in the genera *Colwellia*, *Pseudoalteromonas*, *Shewanella* and *Winogradskyella*	Antibiofilm activity against *Pseudomonas aeruginosa* and *Staphylococcus aureus*	[211]
*Tedania charcoti*, *Haliclonissa verrucosa, Hemigellius pilosus*, Porifera	Strains in the genera *Colwellia*, *Shewanella* and *Winogradskyella*)	Possible application of produced EPSs in the detoxification of metal-contaminated environments and as cryoprotectant	[235]

* If specifically reported. ** PKS, Polyketide synthase; Bcc, *Burkholderia cepacia* complex; MRSA, Methycillin-resistant *Staphylococcus aureus*; EPS, Extracellular Polymeric Substance.

## Data Availability

Not applicable.
